# Factors Influencing Substrate Oxidation During Submaximal Cycling: A Modelling Analysis

**DOI:** 10.1007/s40279-022-01727-7

**Published:** 2022-07-12

**Authors:** Jeffrey A. Rothschild, Andrew E. Kilding, Tom Stewart, Daniel J. Plews

**Affiliations:** Sports Performance Research Institute New Zealand (SPRINZ), Auckland University of Technology, Auckland, New Zealand

## Abstract

**Background:**

Multiple factors influence substrate oxidation during exercise including exercise duration and intensity, sex, and dietary intake before and during exercise. However, the relative influence and interaction between these factors is unclear.

**Objectives:**

Our aim was to investigate factors influencing the respiratory exchange ratio (RER) during continuous exercise and formulate multivariable regression models to determine which factors best explain RER during exercise, as well as their relative influence.

**Methods:**

Data were extracted from 434 studies reporting RER during continuous cycling exercise. General linear mixed-effect models were used to determine relationships between RER and factors purported to influence RER (e.g., exercise duration and intensity, muscle glycogen, dietary intake, age, and sex), and to examine which factors influenced RER, with standardized coefficients used to assess their relative influence.

**Results:**

The RER decreases with exercise duration, dietary fat intake, age, VO_2max_, and percentage of type I muscle fibers, and increases with dietary carbohydrate intake, exercise intensity, male sex, and carbohydrate intake before and during exercise. The modelling could explain up to 59% of the variation in RER, and a model using exclusively easily modified factors (exercise duration and intensity, and dietary intake before and during exercise) could only explain 36% of the variation in RER. Variables with the largest effect on RER were sex, dietary intake, and exercise duration. Among the diet-related factors, daily fat and carbohydrate intake have a larger influence than carbohydrate ingestion during exercise.

**Conclusion:**

Variability in RER during exercise cannot be fully accounted for by models incorporating a range of participant, diet, exercise, and physiological characteristics. To better understand what influences substrate oxidation during exercise further research is required on older subjects and females, and on other factors that could explain additional variability in RER.

**Supplementary Information:**

The online version contains supplementary material available at 10.1007/s40279-022-01727-7.

## Key Points

**Table:** 

Several factors are known to influence substrate oxidation during exercise, but the effect of simultaneously modulating multiple factors on the respiratory exchange ratio (RER) is unclear.
Factors known to influence substrate oxidation during exercise, such as exercise duration and intensity, age, sex, fitness level, muscle glycogen, and daily dietary intake, together explain ~ 59% of the variation in RER during exercise.
The easily measured and easily modifiable factors related to exercise such as exercise duration and intensity, daily macronutrient intake, and pre- and peri-exercise carbohydrate intake, can only explain roughly one-third of the variation in RER during exercise. This suggests most of what dictates RER during exercise cannot be easily controlled on a daily basis.

## Introduction

Energy production during continuous, submaximal exercise comes primarily from the oxidation of fat and carbohydrate. The respiratory exchange ratio (RER) represents an indirect measure of the skeletal muscle respiratory quotient (RQ)—the quantity of CO_2_ produced in relation to O_2_ consumed [1]. The RER can be used to estimate the relative contributions of fat and carbohydrate to energy production with higher values equating to increased carbohydrate reliance and lower values representing increased fat reliance [2]. Several factors are known to influence the RER during exercise including exercise duration [3], exercise intensity [4], training status [5], sex [6], dietary intake [7–9], the pre-exercise meal [10, 11], and carbohydrate ingestion during exercise [3, 12]. However, the relative influence and interaction between these factors is unclear. For example, RER decreases with exercise duration (i.e., increased reliance on fat oxidation), but increases with exercise intensity and carbohydrate intake [13], leaving the net effect on RER unclear when multiple factors are being manipulated. Therefore, a better understanding of the factors influencing RER during exercise is needed.

The ability to effectively oxidize fat for fuel, represented by a lower RER, is important for metabolic health [14] and long-duration exercise performance [15, 16], and many athletes attempt to manipulate substrate oxidation during exercise as part of a periodized nutrition and training plan [17, 18]. However, managing substrate oxidation during exercise is challenged by the influence of both modifiable and non-modifiable factors, which may or may not be easily measured (Table 1). Previous studies have investigated factors influencing substrate oxidation, but none have considered variables often manipulated by athletes such as the duration or intensity of exercise, the pre-exercise meal, or carbohydrate ingestion during exercise. Goedecke et al. [19] found the most important factors influencing RER during endurance exercise were mitochondrial enzyme activity, muscle glycogen and triglyceride concentrations, dietary fat intake, training volume, and free fatty acid concentrations, which collectively explained 42–56% of the variation in RER during exercise. Distinct from RER, others have studied the determinants of maximal fat oxidation rates and found 34–79% of the variance was related to factors such as maximal oxygen consumption (*V*O_2max_), sex, body composition, physical activity level, dietary macronutrient intake, resting fat oxidation, and fasting duration [20–24]. To our knowledge, the relative influence of the modifiable, easily measured factors influencing RER during exercise (e.g., dietary intake before and during exercise, exercise duration, and exercise intensity) has yet to be established. Using multivariable regression models, it would be possible to account for multiple factors influencing RER during exercise and predict the response under various circumstances. Therefore, the purpose of this analysis was to investigate factors influencing the RER during cycling exercise and formulate regression models to determine which factors best explain RER during exercise, their relative influence, and the result of multiple variables being modulated simultaneously. To this end, we performed the largest pooled analyses to date (~ 3400 RER observations) of studies examining substrate oxidation during exercise and provide novel insight into the factors influencing fuel selection during endurance exercise.

**Table 1:** Factors influencing respiratory exchange ratio (RER) during exercise and ease of day-to-day modification and measurement

	Easily measured	Not easily measured
Easily modified	Exercise duration [3, 12] Exercise intensity [5] Dietary CHO and fat intake [25] Pre-exercise CHO intake [26] CHO during exercise [3, 12] Type of CHO consumed [27] Energy balance [28] Pre-exercise meal timing [29] Cycling cadence [30]	Muscle glycogen [19, 25] Muscle triglycerides [19] Hydration status [31] Glycemic index [32]
Not easily modified	Age [33] Training age [34] Training volume [19] Sex [6] Menstrual phase [35] and status [36] Fitness level/*V*O_2max_ [5, 37] Ventilatory/lactate thresholds [34] Plasma lactate [19] Fasting/resting RER [19] Body composition [38] Environmental temperature [39] Altitude [40]	Type I muscle fiber percentage [19, 30] Mitochondrial enzymes/proteins [19] Plasma free fatty acids [19] Genetic variation [41] Habitual physical activity levels [22] Catecholamines [42]

*CHO* carbohydrate, *VO*_*2max*_ maximal oxygen consumption

## Methods

### Eligibility Criteria

#### Inclusion Criteria

Studies of healthy adult (> 18 years of age) humans were included for analysis. Only studies using two-legged cycling exercise were included, due to differences in substrate utilization between cycling and running [43, 44]. Cycling had to be continuous, for at least 5 min in duration and performed at a single exercise intensity. If there were changes in exercise intensity, only the first intensity was included [45–49]. Studies must have been performed in a normoxic, temperate environment (15–25 °C), and subjects must not have performed any exercise within 12 h of the trial due to the influence of prior exercise on substrate oxidation [50, 51].

#### Exclusion Criteria

Participants could have any physical activity level, but people with metabolic disorders were excluded. Children and teenagers were excluded due to differences in substrate utilization compared with adults [52]. Studies using a pre-exercise fasting period > 15 h were excluded to maximize generalizability and practical application. Both fixed-duration and time-to-exhaustion trials were included, but time trials were excluded due to the variability of pacing and intensity.

### Search Strategy

A PubMed search was performed on 30 April 2021 and included all publication years up to and including the date the search was conducted, using the following terms: (cycling OR endurance OR exercise OR "prolonged exercise") AND (carbohydrate) AND ("fat oxidation" OR metabolism OR "muscle glycogen" OR "oxygen uptake" OR "substrate oxidation" OR "substrate utilization" OR "carbohydrate oxidation" OR "energy expenditure" OR "skeletal muscle" OR "substrate metabolism” OR “respiratory exchange ratio”) AND (clinical trial [Filter] OR randomized controlled trial [Filter] NOT (diabetes) NOT (running) NOT (treadmill) NOT (resistance). In addition, the reference sections of studies included in this analysis were searched. The titles, abstracts, and full-text articles were independently screened by the lead author (JR). A second author (DJP) was consulted if there was uncertainty about article eligibility. The rationale for excluding articles was documented.

### Data Extraction

The following data were extracted from papers meeting the above criteria: RER, duration (min), exercise intensity (%*V*O_2max_), sex (% female subjects), daily carbohydrate and fat intake (as percentage of energy intake, and as grams ingested in total and relative to body mass), carbohydrate and fat intake 4 h pre-exercise (g), number of minutes before exercise food was consumed, carbohydrate ingestion during exercise (hourly intake rate as well as the drink composition as percentage glucose, fructose, and other carbohydrate sources), starting and ending muscle glycogen levels (mmol·kg^−1^ dry mass), age (y), training age (y), percentage of type I muscle fibers, body mass index (BMI), *V*O_2max_ (mL·kg·min^−1^), glycemic index of the pre-exercise meal, sample size, and study ID.

The RER had to be reported at multiple time points for exercise lasting longer than 30 min, and/or consist of no more than a 30-min average value. When reported at multiple time points each value was recorded as a separate data point. Substrate oxidation reported in grams per minute was converted to an RER value using equations from Jeukendrup and Wallis [1]. Exercise intensity had to be reported or be able to be calculated as a percentage of *V*O_2max_. Sex was analyzed as a categorical variable, with the study population considered “male” or “female” if ≥ 70% of subjects were of one sex, and “mixed” if the split was 30–70%. A categorical variable was created for type of ingestion during exercise and included carbohydrate, fat, protein, water, carbohydrate and protein, and carbohydrate and fat.

Muscle glycogen concentrations before, during, and after exercise were recorded when determined using whole muscle (not fiber-type specific), from muscle biopsies (excluding non-invasive measures such as magnetic resonance spectroscopy). Biopsies must have been performed before and within 30-min post exercise. For studies that took a resting biopsy before providing pre-exercise carbohydrate, starting glycogen concentrations were only recorded for the placebo/control group but ending glycogen levels were recorded for all groups [53, 54]. Studies that depleted glycogen in only one leg prior to an exercise trial were excluded. The conversion factors of Areta and Hopkins [55] were used when glycogen values were reported in units other than mmol·kg^−1^ dry mass.

When studies included multiple groups, data were used if the interventions included factors that were accounted for in the analysis (e.g., differences in carbohydrate ingestion, exercise intensity, or sex) but only the control/placebo groups were used if the intervention arm included a variable not analyzed such as the use of heparin [56, 57], estrogen [58, 59], glucose infusion [60, 61], caffeine [57], alcohol [62], or various dietary supplements [47, 63–74]. Studies that provided protein or fat during exercise were included due to the minimal influence of protein [75–78] or fat [11, 79–84] ingestion on RER, although it is possible that RER values may be less reliable under conditions of increased gluconeogenesis, lipogenesis, or ketogenesis [1, 85]. Interventions that used 5–6 days of a high-fat diet followed by 24-h carbohydrate restoration were excluded due to persisting effects of the high-fat diet on RER [86–91].

### Statistical Approach

To find the factors that best explain RER as well as focus on the influence of modifiable and easily measured variables, multiple mixed-effect models were created. All available variables were initially modelled, before focusing specifically on easily measured variables (i.e., excluding glycogen concentration and muscle fiber type but including age, sex, and *V*O_2max_), and those that are both easily measured and easily modified, due to the strong emphasis placed by athletes and coaches on the influence of carbohydrate consumption on fat oxidation [18, 92].

Univariable regression analysis was first performed between variables of interest and RER, and the best-fit regression line (linear or polynomial) was established using the likelihood ratio test. Because data points were collected at multiple time points during exercise in each study, the assumption of independence of residuals is violated. Therefore, we built general linear mixed-effect models to examine how each individual factor was related to RER, specifying study ID as a random intercept using the *lme4* R package [93], and report the marginal R^2^, which describes the proportion of variance explained by only the fixed effect.

We then built general linear mixed-effect models to examine which factors were related to RER, with study ID again specified as a random intercept. The following fixed effects were tested: starting muscle glycogen, end-exercise muscle glycogen, exercise duration (min), exercise intensity (%*V*O_2max_), daily carbohydrate and fat intake (as percentage of energy intake, total g, and g per kg body mass), carbohydrate and fat intake within 4 h of exercise (g), minutes before exercise carbohydrate was consumed, carbohydrate intake during exercise (g h^−1^), percentage of glucose and fructose in drinks ingested during exercise, ingestion type during exercise (as a categorical variable), fitness level (*V*O_2max_), age, sex, and percentage of type I muscle fibers. Interactions between RER and other fixed effects were explored, and the optimal, best-fitting model was decided based on the likelihood ratio test. The fit of each model was checked by visualizing the Q–Q and other residual plots to ensure approximate residual normality and heteroscedasticity, and outliers were removed based on a composite outlier score using the *performance* R package. Multicollinearity was assessed using the variance inflation factor (VIF), with values > 5 used to indicate excessive collinearity [94]. Model fit is reported as marginal *R*^2^ as well as conditional R^2^, which describes the proportion of variance explained by both the fixed and random effects [95], and root mean square error (RMSE). Estimated means were calculated using the *emmeans* package [96]. Descriptive statistics are provided as mean ± SD. All analyses were carried out with R version 4.0.3 (The R foundation for Statistical Computing, Vienna, Austria), with the level of significance set at *p* < 0.05.

## Results

### Included Studies

The database search yielded a total of 8052 results. Following the removal of 63 duplicates, 7989 titles and abstracts were screened. A total of 784 full-text articles were screened for eligibility, and data were extracted from 434 studies that met the inclusion criteria. Due to the large influence of daily macronutrient intake on RER, all multivariable models included daily dietary intake as a variable. Consequently, studies not reporting dietary intake during the 24 h prior to exercise were excluded from all multivariable models. Therefore, the univariable analysis includes data from 434 studies (3498 RER observations) whereas the multivariable models contained data from 106 studies, which included 1,221 participants (21.2% female, mean age 26.5 ± 5.5 years, range 19–52 years, BMI 23.2 ± 1.3 kg/m^2^, *V*O_2max_ 53.6 ± 9.6 mL kg^−1^ min^−1^) and 1104 RER observations, noted fully in the Online Supplemental Material (OSM).

### Correlations

**Fig. 1:**
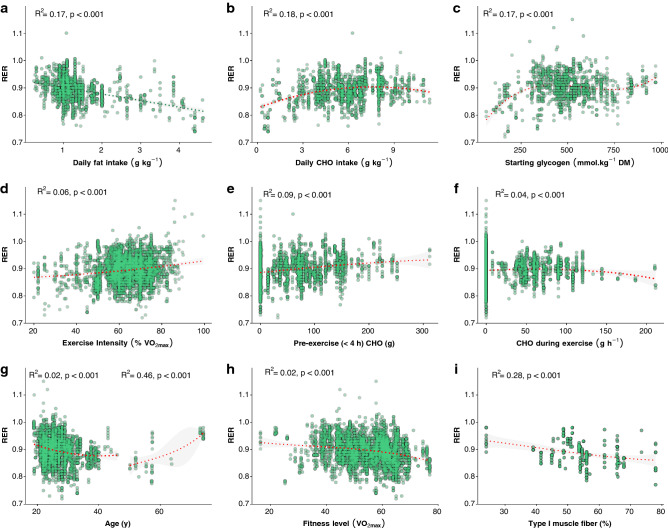
Relationships between respiratory exchange ratio (RER) and factors influencing RER. Best-fit regression lines based on univariable mixed effect models are shown, with fit indicated as *R*^2^. Best-fit lines using linear regression are shown in green, best-fit lines using polynomial regression and are shown in red. Panel (g) is separated by the natural gap in the data of mean age > or < 50 years. Shaded areas represent 95% confidence intervals. *CHO* carbohydrate, *DM* dry mass

**Fig. 2:**
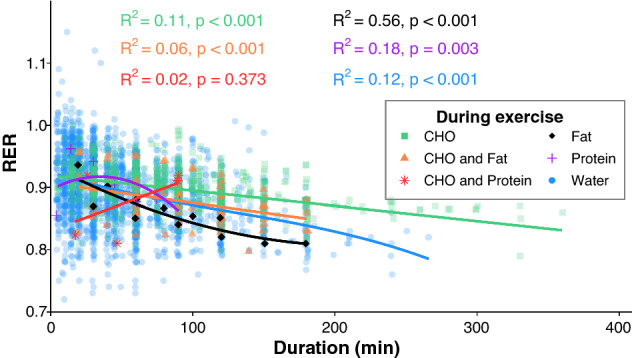
Relationship between respiratory exchange ratio (RER) and exercise duration separated by type of ingestion during exercise. Best-fit regression lines based on univariable mixed effects models are shown for each ingestion type during exercise, with fit indicated as *R*^2^ and *p* value. *CHO* carbohydrate

Relationships between RER and the primary factors influencing it are shown in Fig. 1. Regression lines and R^2^ values are shown, with the best-fit lines for daily carbohydrate intake, starting glycogen, pre-exercise carbohydrate intake, carbohydrate ingestion during exercise, age, percentage of type I muscle fibers, and exercise intensity being curvilinear. For age, the available data shows a negative relationship with RER below age 50 years and positive relationship above age 50 years (Fig. 1g). However, data points above age 52 years could not be used in the multivariable modelling due to unreported dietary intake, therefore the models only reflect a negative relationship. A significant positive relationship was also found between RER and the glycemic index of the pre-exercise meal (*R*^2^ = 0.08, *p* < 0.001), but the small number of studies reporting this measure (*n* = 10) precluded its use in the models and thus is not shown. The relationship between exercise duration and RER is shown in Fig. 2, stratified by the type of ingestion during exercise. Limited comparisons can be made relating to the effect of each type of nutrient ingestion due to the limited number of data points for some of the variations, but are shown to demonstrate the available data and observable trends.

### Models

**Fig. 3:**
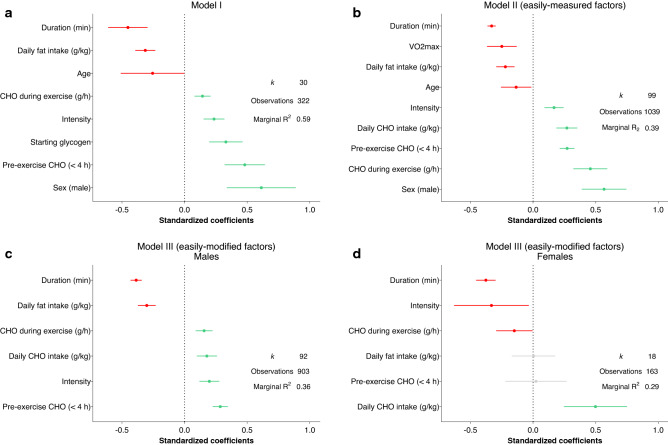
Standardized coefficients for model parameters. These figures depict the relative influence each variable has on respiratory exchange ratio (RER) during exercise. For clarity, interaction effects are not shown. Intensity is exercise intensity as %*V*O_2max_. Marginal *R*^2^ denotes the variance explained by the fixed factors alone. *CHO* carbohydrate, *k* number of studies included. Green bars represent factors that increase RER, red bars indicate factors that decrease RER, lines indicate 95% confidence intervals

The best fitting models are shown in Table 2, with the fixed effects explaining up to 59% of the variation in RER. The models show RER decreases with exercise duration, dietary fat intake, age, and *V*O_2max_, and increases with dietary carbohydrate intake, exercise intensity, male sex, and carbohydrate intake before and during exercise. Model I, which explains the greatest amount of variance in RER, includes 322 RER observations. Models II (easily measured factors) and III (easily modified factors, for males and females separately) contain more observations but explain a lower proportion of the variation in RER. To visualize the relative influence of each variable, standardized model coefficients are shown in Fig. 3. Additionally, a model that included the percentage of type I muscle fibers was also made, which explained 56% of the variation in RER, and included daily carbohydrate intake, carbohydrate ingestion during exercise, and exercise intensity (not shown). However, other factors such as sex could not be included in the model with muscle fiber type due to the completeness of available data. Overall, variables with the largest influence on RER are sex and exercise duration, and among the diet-related factors, daily fat and carbohydrate intake has a larger influence than carbohydrate ingestion during exercise.

**Table 2:** Models to explain variation in respiratory exchange ratio (RER) during submaximal cycling

Variable	Model I	Model II (easily measured factors only)	Model III (easily modified factors only—males)	Model III (easily modified factors only—females)
Starting glycogen (mmol kg^−1^ dry mass)	0.0001 (< 0.001)			
Daily CHO intake (g kg^−1^ day^−1^)		0.0058 (< 0.001)	0.0038 (< 0.001)	0.0105 (< 0.001)
Daily fat intake (g kg^−1^ day^−1^)	− 0.0179 (< 0.001)	− 0.0129 (< 0.001)	− 0.0172 (< 0.001)	0.0004 (0.962)
Pre− exercise (< 4 h) CHO intake (g)	0.0004 (< 0.001)	0.0002 (< 0.001)	0.0002 (< 0.001)	1.3e−05 (0.852)
CHO during exercise (g h^−1^)	0.0003 (< 0.001)	0.0007 (< 0.001)	0.0002 (< 0.001)	− 0.0003 (0.043)
Duration (min)	− 0.0006 (< 0.001)	− 0.0004 (< 0.001)	− 0.0004 (< 0.001)	− 0.0005 (< 0.001)
Intensity (%*V*O_2max_)	0.0015 (< 0.001)	0.0008 (< 0.001)	0.001 (< 0.001)	− 0.0014 (0.030)
Sex (male)	0.0158 (0.104)	0.0328 (< 0.001)		
Age (y)	− 0.0039 (0.037)	− 0.0012 (0.030)		
Fitness level (*V*O_2max_)		− 0.0013 (< 0.001)		
Duration × starting glycogen (1)^a^	− 0.0003 (0.757)			
Duration × starting glycogen (2)^a^	− 0.0021 (< 0.001)			
Duration × CHO during exercise		6.0e−07 (0.191)	4.5e−07 (0.119)	1.4e−05 (< 0.001)
Sex × CHO during exercise		− 0.0004 (< 0.001)		
Duration × sex	0.0003 (0.024)			
Intercept	0.859	0.912	0.843	0.924
Marginal *R*^2^	0.59	0.39	0.36	0.29
Conditional *R*^2^	0.90	0.85	0.86	0.95
RMSE	0.018	0.018	0.018	0.013
*k*	30	99	92	18
Observations	322	1039	903	163

Linear coefficients, their corresponding *p* values (in parentheses), marginal *R*^2^ (variance explained by the fixed factors alone), conditional *R*^2^ (variance explained by both the fixed and random effects), root mean square error (RMSE), number of studies (*k*), and number of observations included in the best-fitting linear mixed models to explain RER during exercise using different factors

^a^(1) and (2) refer to the polynomial terms for starting glycogen

### Predictions

**Fig. 4:**
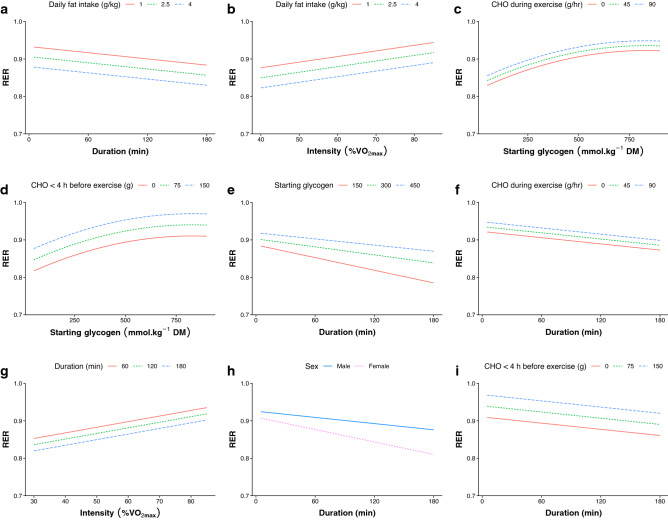
Model predictions showing the interactions between pairs of variables across a range of values, based on Model I. For example, in (**a**) it can be surmised that the respiratory exchange ratio (RER) at 60 min, when consuming a diet consisting of 2.5 g kg^−1^ of fat per day, would be similar to the RER at 180 min after consuming 1 g kg^−1^ fat per day. Panel (**a**) also shows the predicted RER across different time points (0–180 min) following a given dietary fat intake. Steeper slopes indicate a larger effect of the variable shown on the *x*-axis, and greater space between lines indicates a larger effect of the variable being plotted. *DM* dry mass

**Fig. 5:**
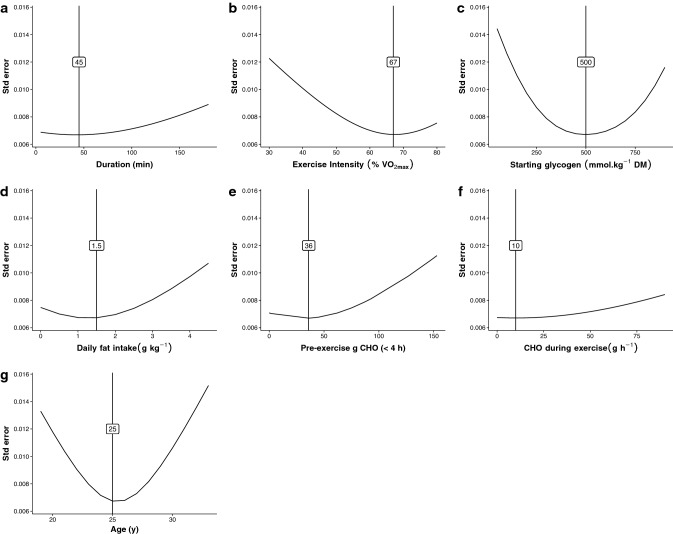
Model I standard errors. This figure depicts the level of certainty in terms of what we currently know about the effects of these factors on respiratory exchange ratio (RER) (i.e., we can be more confident in the relationship between these factors and RER, when the standard error is lower). The number denoted in each panel indicates the value with the lowest standard error. *DM* dry mass

To visualize the interaction and convergence of multiple factors influencing RER during exercise, estimated marginal means from Model I are shown in Fig. 4 in various combinations. The standard error of the estimated means, across the range of values for each fixed effect in Model I, are shown in Fig. 5. This represents the confidence in the predicted values shown in Fig. 4, and how this confidence changes depending on the value of the fixed effect. When more data points are involved in the calculation of the mean, it tends to lead to smaller standard errors. Therefore, this provides an indication of where the most research has been performed, for each of the variables studied. The value with the lowest standard error is shown in each panel. For the interested reader, an online app has been created to allow exploration of the data and predict RER based on the data used in this analysis [97].

## Discussion

The purpose of this analysis was to investigate factors influencing the RER during cycling exercise, understand their relative influence, and determine how RER is affected when multiple variables are modulated simultaneously. This knowledge is important for athletes and coaches/practitioners who wish to manipulate substrate oxidation during exercise. The key findings are that exercise duration and intensity, age, sex, fitness level, muscle glycogen, and daily dietary intake together explain ~ 60% of the variation in RER during exercise, indicating a large influence of additional factors, and that daily dietary intake has a larger influence on RER than carbohydrate ingested during exercise. Additionally, the biggest relative determinants of RER during exercise are sex and exercise duration, with pre-exercise carbohydrate intake and daily fat intake also identified as main determinants.

To our knowledge, this is the first large-scale attempt to analyze the determinants of substrate oxidation during exercise using modifiable factors such as exercise intensity, exercise duration, and dietary intake before and during exercise. Goedecke et al. [19] found mitochondrial enzyme activity, muscle glycogen and triglyceride concentrations, dietary fat intake, training volume, and free fatty acid concentrations collectively explained 42–56% of the variation in RER during exercise. Others have studied the determinants of maximal fat oxidation rates and found 34–79% of the variance was related to factors such as *V*O_2max_, sex, body composition, physical activity level, 4-day dietary intake, resting fat oxidation, and fasting duration [20–24]. However, these studies did not consider aspects that are routinely modulated by athletes such as the exercise duration or intensity, or pre- and peri-exercise carbohydrate intake.

### Modifiable Factors

#### Diet and Starting Glycogen

Our analysis highlights the influence of daily macronutrient intake on RER during exercise, particularly dietary fat intake. It is challenging to distinguish between the influence of dietary carbohydrate and dietary fat intake, as they are often manipulated together. However, there are several reasons why it can be speculated from our findings that daily dietary fat intake may have a greater influence than dietary carbohydrate intake on RER during exercise. The best-fitting model for RER (Model I) included dietary fat intake and was not significantly improved by the inclusion of dietary carbohydrate intake. Furthermore, RER during exercise was decreased more following 5 days of a high-fat diet, compared with a high-protein diet, when dietary carbohydrate was clamped below 20% of energy intake for both groups [91]. Although a portion of the increased dietary protein intake in that study was likely converted to glucose via gluconeogenesis [98], a short-term increase in dietary fat can decrease the amount of the active form of pyruvate dehydrogenase (PDH) [99], which is the rate-limiting enzyme in carbohydrate metabolism. The rate of glycolysis appears to play a central role in the regulation of fatty acid oxidation [100], and the downregulation of PDH observed following 5 days of a high-fat diet is not offset by 1 day of high-carbohydrate intake [90]. Taken together, both dietary fat and dietary carbohydrate influence RER during exercise, but daily dietary fat intake may have a stronger influence.

Bivariate correlations between daily carbohydrate intake and RER suggest a curvilinear relationship (Fig. 1b), which could imply a diminishing influence of dietary carbohydrate on RER past a certain threshold (~ 4 g kg^−1^). This is a finding that could be explored in future research to determine if there is a threshold for carbohydrate oxidation to occur based on demand, without being influenced by limitations from an undersupply of carbohydrate, and to investigate the shape of the relationship (i.e., linear or curvilinear) between dietary carbohydrate intake and PDH activity. However, this could also simply reflect fewer data points in the upper ranges of daily carbohydrate intake, particularly because the multivariable regression models were not improved when including the polynomial term (implying other variables could sufficiently explain the differences in RER).

A non-linear relationship was also found between starting muscle glycogen and RER (Fig. 1c), suggesting the influence of glycogen on RER could differ based on concentration, or could again simply reflect fewer data points at the lower and upper ranges. However, unlike daily carbohydrate intake, there was significant model improvement when using the polynomial term and a significant duration*glycogen interaction, which makes sense mechanistically because the rate of glycogen breakdown varies with initial concentration and is reduced with exercise duration [55]. The correlations shown in Fig. 1c and the model predictions shown in Fig. 4c and 4d both suggest a leveling off of the influence of starting muscle glycogen on RER; however, the precise breakpoint where this occurs should be investigated in future research.

Dietary carbohydrate intake increases muscle glycogen concentration. Undertaking exercise with higher levels of muscle glycogen can increase RER by increasing muscle glycogenolysis, but does not influence exogenous carbohydrate oxidation rates [25, 101]. Although it could be assumed the influence of the daily carbohydrate intake is solely due to changes in starting muscle glycogen concentrations, other factors are likely involved such as changes in enzyme activity and/or gene expression related to carbohydrate and lipid metabolism seen following short-term high- and low-carbohydrate diets [102–104]. This notion is supported by the persisting effects on RER from 5 to 6 days of a high-fat diet followed by 24-h carbohydrate restoration, despite similar starting muscle glycogen concentrations [86, 87, 90]. Changes in substrate utilization following two weeks of a high-fat diet have also persisted through 3 days of a high-carbohydrate diet [105], but 1 week of a high-carbohydrate diet abolished the increases in fat oxidation observed following a 7-wk high-fat diet [9], indicating the approximate time-decay for changes in enzyme activity.

Studies in this analysis reported dietary intake for an average of 4.5 ± 7.4 (range 1–49) days, but the number of days reported did not influence the models. It seems unlikely that including only longer-term (habitual) dietary intake would have significantly changed the findings, as the decreased RER observed on a low-carbohydrate diet was not different when tested after 2 days or 2 weeks [106], or after 5 days and 15 days [107]. However, diets with extreme short-term variation (e.g., 5–6 days of a high-fat diet followed by 24-h carbohydrate restoration [86–91]) are known to have lingering effects on RER and were therefore excluded from the analysis. Accuracy of dietary reporting is a noteworthy concern when participants are in a free-living situation [108], and likely varies based on the method of dietary control. Some studies simply measured participants’ habitual dietary intake [62, 109], whereas others provided short-term [12, 91, 110] or longer-term [9] standardized diets to study participants. Data were extracted from 400+ studies but the majority were not included in the models because they did not report dietary intake, instead reporting that participants were instructed to note and repeat their 24-h dietary intake before each study visit. It would be beneficial for future studies analyzing substrate oxidation during exercise to report daily macronutrient intake.

#### Pre-Exercise Meal

Ingesting carbohydrate before exercise increases plasma glucose and insulin levels, reduces hepatic glucose output, and increases skeletal muscle glucose uptake during exercise [111]. This can lower fat oxidation by decreasing plasma free fatty acid availability via insulin-mediated inhibition of lipolysis [112], and also by inhibiting fat oxidation within the muscle due to an increased glycolytic flux [113]. Accordingly, our models highlight the strong influence of pre-exercise carbohydrate on RER during exercise (Figs. 3, 4). In an attempt to increase fat utilization (i.e., decrease RER) during exercise, many endurance athletes train in the overnight-fasted state [18], although evidence on whether the repeated practice of fasted-state training translates to longer-term increases in fat oxidation capacity remains equivocal [13]. More research, using endurance-trained subjects, is needed to determine whether longer-term fasted training increases fat oxidation during continuous exercise, particularly when tested in the carbohydrate-fed state.

There are several aspects related to the pre-exercise meal that may exert influence on RER but were not influential in the final models, likely because of not enough data points. The effect of glycemic index on RER has been equivocal, with lower-index meals resulting in lower [32], higher [114], or similar [115, 116] RER values during exercise, but only three studies meeting the inclusion criteria for our analysis also reported daily dietary intake. The size and timing of the pre-exercise meal may also influence RER, with a higher RER observed following larger meals eaten farther in advance of exercise [13, 29]. Pre-exercise protein ingestion has resulted in similar RER values to those in fasted-state exercise [117], although this may be influenced by the type of protein and degree of hydrolyzation [118], and we found no influence of dietary fat in the pre-exercise meal on RER. Therefore, in our analysis the pre-exercise meal was quantified only by carbohydrate intake, meaning pre-exercise protein and/or fat ingestion, in the absence of carbohydrate, was analyzed in the same way as fasted-state training. However, there is opportunity for future research to further explore pre-exercise protein and its effects on substrate oxidation, including the type of protein, its effects on gluconeogenesis and urea formation [119], and if its influence may be intensity-dependent [10].

#### Peri-Exercise Intake

Our analysis revealed a small yet significant influence of carbohydrate intake during exercise on RER. Carbohydrate ingestion during exercise maintains blood glucose levels, carbohydrate oxidation, and RER, and prevents the depletion of liver, but not muscle, glycogen [120–123]. Increasing the rate of carbohydrate ingestion during exercise decreases hepatic glucose output and increases the contribution of exogenous carbohydrate oxidation to total energy contribution in a dose–response manner [124], at least up to the point where gastrointestinal transport of sugars becomes saturated [125]. However, differences in ingestion rate are not always reflected as differences in RER [12, 124, 126]. The RER is most likely to be influenced after ~ 1.5 to 2 h of exercise as endogenous carbohydrate availability declines [3], but differences in RER between carbohydrate and placebo ingestion can be seen earlier in exercise, particularly with very high carbohydrate ingestion [127]. The type of carbohydrate ingested may influence rates of endogenous and exogenous carbohydrate oxidation, but total carbohydrate oxidation (and RER) appears less affected [128–131]. However, future research should examine the differences in carbohydrate type in the context of high (> 100 g h^−1^) ingestion rates, as contrasting findings have been reported [123, 131]. Carbohydrate ingestion may fail to influence RER when exercise intensity is high [132], and/or in untrained participants [133], as RER may already be elevated in these circumstances. However, our models were not significantly improved by interaction effects between carbohydrate ingestion during exercise and either exercise intensity (*p* = 0.119) or *V*O_2max_ (*p* = 0.179).

The influence of protein and fat ingestion during exercise was explored, but the models were not improved by inclusion of the type of peri-exercise nutrition. Although most studies have reported a minimal influence of protein [75–78] or fat [11, 79–84] ingestion on RER, the RER is typically interpreted based on the assumption of negligible protein oxidation. This assumption could be invalidated in the context of protein ingestion before or during exercise due to increased gluconeogenesis, which could decrease RER irrespective of any change in fat oxidation rate via transfer of the amino group to the urea cycle [1], or by stimulating glucagon secretion, which promotes gluconeogenesis and increases fat oxidation [134]. Some evidence suggests high dietary protein intake [98] or protein ingestion during fasted exercise [135] may have a notable effect on gluconeogenesis, and could explain why protein ingestion before or during exercise has been reported to increase fat oxidation in runners [109, 118].

It is also possible that fat intake during exercise, often provided in the form of medium chain triglycerides (MCT), can influence substrate oxidation via an increase in ketogenesis [80, 83]. Unlike long-chain fatty acids, MCTs are rapidly absorbed into the hepatic portal system and transported into the mitochondria independent of transporter proteins [136]. Although the studies included in this analysis have not found an effect of MCT when ingested with carbohydrate, this may be related to the amount provided and the specific exercise context. Reduced carbohydrate oxidation with combined MCT and carbohydrate ingestion compared with carbohydrate ingestion alone during exercise has been observed in one study, but dietary intake was not reported (and thus not included in the modelling) [137].

A broad look at the influence of various peri-exercise nutrition options can be seen in Fig. 2, but due to the drastically different number of data points for each condition, limited conclusions can be drawn from comparison. Future studies could further explore the influence of these macronutrient combinations on substrate oxidation during exercise, particularly related to protein and/or ketone oxidation.

#### Exercise Duration and Intensity

It is well established that the RER increases with exercise intensity and decreases with exercise duration [4, 5, 138]. A novel finding of this analysis is that the duration of exercise likely exerts a larger influence on RER than the intensity of exercise, shown by the standardized model coefficients (Fig. 3). However, the influence of exercise duration can only be predicted using these models for activities less than ~ 3 h, as the longest time point reported for the studies included in Model I was 180 min. The longest study that was included in Model III was 360 min [139], but because not all modeled variables were reported, it could not be included in Models I–II. Based on Fig. 2, which includes all extracted data, it appears RER may level off around 180 min and remain higher when fed carbohydrate compared with water, but further research is needed to confirm this.

Exercise intensity was analyzed as a percentage of *V*O_2max_ because that is the most widely reported unit in exercise science. This can be problematic because substrate use can vary greatly at a given percentage of VO_2max_ depending on whether someone has a high or low lactate threshold [34, 140]. Therefore, the use of lactate or ventilatory thresholds could be a better reference for exercise intensity [141], and/or could also be included as a model variable in future regression analysis. Although RER should not be used as an index of substrate utilization above 75% *V*O_2max_ [1], we included all available data points in the models because we modelled RER and not substrate oxidation in absolute values (grams per minute, etc.). This means inferences pertaining to absolute values of substrate oxidation should not be made using this data for exercise intensities > 75% *V*O_2max_.

#### Additional Modifiable Factors

Total daily energy intake can also play a role in the RER response during exercise, particularly in the context of low energy availability. Low energy availability describes a mismatch between an athlete’s energy intake (diet) and the energy expended in exercise, leaving inadequate energy to support the functions required by the body to maintain optimal health and performance [142]. Endurance athletes with high training volumes are at risk of chronically low energy availability, which can reduce resting metabolic rate and influence the normal metabolic hormonal milieu that may alter the RER response to exercise, as well as influence RER via changes in muscle glycogen concentration [143]. The majority of studies in this analysis standardized dietary intake and had study participants rest before exercise trials, reducing the ability to investigate low energy intake in the models. Future studies investigating variability in RER, as well as for practical application of these findings, should consider the influence of energy availability as a factor that could reduce RER during exercise.

### Non-Modifiable Factors

#### Sex

Sex is known to influence substrate oxidation and was among the strongest influences on RER in our models. Along with hormonal differences, sex-based differences in lipid storage within the muscle and liver, and in the percentage of type I muscle fibers, can help explain differences in substrate oxidation during exercise [144]. The RER is generally lower for women during submaximal exercise [6], however, this is not a universal finding [19, 145–148]. Divergent findings may be related to carbohydrate intake before and during exercise, and/or the duration of exercise. In several studies that did not find sex differences in substrate oxidation the dietary carbohydrate intake was lower in males [19, 146], whereas studies controlling dietary intake have often [149, 150], although not always [147], found a lower RER in females compared with males. Carbohydrate ingestion during exercise can also attenuate sex differences in RER [151, 152].

Our modelling revealed significant interactions between sex and exercise duration (Model I) and between sex and carbohydrate ingestion during exercise (Model II). However, a sex × carbohydrate ingestion during exercise interaction could not be explored in Model I because there were no studies in the analysis that provided exogenous carbohydrate to female subjects while also reporting muscle glycogen concentrations. Although women typically have a greater percentage of type I muscle fibers than men [144], sex could not be included in a model with fiber type percentage due to only one study reporting fiber-type percentage in females [153]. Sex differences in RER would more likely be observed at lower exercise intensities and diminish as the intensity increases and the RER approaches 1.0 [154], but a sex × intensity interaction was left out of the models due to excessive collinearity in the data.

A potential limitation in our analysis is not controlling for menstrual cycle. A lower RER has been reported in the luteal, compared with follicular, phase of the menstrual cycle [155]; however, these differences are obscured with carbohydrate ingestion during exercise [156]. Others have found no influence of menstrual phase during 60–75 min of cycling [157–159], or an effect of menstrual phase that only became apparent after 75–90 min of cycling [160]. Increased estrogen concentrations suppress gluconeogenesis [161] and promote increased lipid availability and increased fat oxidation capacity [162], which could decrease RER in the late-follicular phase and luteal phases, although this influence may be antagonized by progesterone making the net effect in the luteal phase dependent on the relative effects of both ovarian hormones [163]. These differences could also be related to muscle glycogen at the start of exercise, which may be lower in the mid-follicular, compared with mid-luteal, phase when on a normal/mixed diet (~ 5 g/kg carbohydrate), but is not different on a high-carbohydrate (8.4 g/kg) diet [157]. However, muscle glycogen sparing during exercise has also been observed in the luteal compared with the follicular phase [164]. It is therefore possible that combining all female cohorts together may be introducing some error in the models, but these effects are likely attenuated in the context of other factors such as daily carbohydrate intake, pre-exercise carbohydrate intake, and carbohydrate ingestion during exercise.

#### Fitness level/*V*O_2max_

It is well established that trained athletes have a lower RER than untrained subjects at a given exercise intensity [5, 37, 165], due to training-induced increases in the ability to oxidize fatty acids, thus sparing muscle glycogen and blood glucose during exercise [166]. A significant negative relationship between *V*O_2max_ and RER was confirmed in our analysis when considering all 3,498 available data points (Fig. 1h), and the 1039 data points included in Model II (*r* = − 0.11, *p* = 0.001), but not among the 322 data points included in Model I (*r* = 0.02, *p* = 0.736). Because the only difference between studies included in a model was whether all factors were reported, and mean *V*O_2max_ was similar across models, the lack of inclusion into Model I is likely related to the comparatively small number of observations.

As an indicator of fitness level/training status, *V*O_2max_ has been used to distinguish between trained and untrained participants [147, 167], and is known to increase with endurance training [168]. However, *V*O_2max_ alone may insufficiently account for short-term training-induced adaptations. Despite negligible changes in *V*O_2max_, changes in RER during exercise can be seen following just 7–10 days of training [169, 170]. Furthermore, testing protocols vary and may underestimate someone’s true *V*O_2max_ [171], thus influencing both the relative exercise intensity and our assessment of fitness level. As an alternative measure of fitness status, training age (i.e., number of years performing regular endurance training) could help to explain some of the variability in RER during exercise but was not included in the models due to the limited number of studies reporting this value. An increased training age could be expected to accompany longer-term training adaptations such as an increased lactate threshold and/or higher percentage of type I muscle fibers [34]. Taken together, fitness level, most easily quantified as VO_2max_, likely has a small yet significant negative influence on RER, but other factors may be more predictive of RER during exercise.

#### Age

Despite the inclusion criteria being open to any studies using adults over the age of 18 years, the mean age of the study participants included in the models was 26.5 ± 5.5 (range 19–52) years, with the oldest mean participant age in Model I just 33 years and the oldest group of females just 25 years. Older subjects have been studied [172–174] and are included in Fig. 1g, but those studies did not report pre-trial dietary intake and were therefore excluded from the models. Analysis of the relationship between age and RER suggests the potential for a U-shaped curve, with RER decreasing until middle age and increasing thereafter (Fig. 1g). It has been reported that the training-induced increases in maximal fat oxidation rate may be attenuated with aging [175]. This could be related to the decreased mitochondrial oxidative capacity observed in older humans [176–178], and along with an increased glycogen reliance [174] suggest an upward shift in RER during exercise in older individuals that could not be detected in the models. Studies reporting RER during exercise at the same relative intensity between older and younger subjects have been equivocal, and the effects may differ with training status [33, 179, 180]. However, these studies did not control for diet, limiting the conclusions that can be drawn. Adding to the complexity, comparisons between younger and older subjects can be made based on either the same absolute or relative (%*V*O_2max_) intensity, yet the ventilatory thresholds occur at a higher percentage of *V*O_2max_ in trained older cyclists [181]. Although women under 45 years typically have a higher relative rate of maximal fat oxidation compared to men, these differences are not apparent after age 45 [36], possibly related to post-menopausal status characterized by low estrogen concentrations, higher circulating levels of follicle-stimulating hormone, and decreased lean body mass [182]. This suggests the potential for an age × sex interaction that cannot be accounted for in the modelling. Therefore, the findings that RER decreases with age may not translate to older (> 45 years) adults.

#### Fiber Type

We created a model to investigate the effects of muscle fiber type percentage, despite the small number of data points, because of the mechanistic potential to influence RER, and a significant influence of fiber type percentage was found. An increasing percentage of type I muscle fibers would be expected to predispose someone towards a lower RER at rest and during exercise, due to the differences in reliance on oxidative phosphorylation between types I and II muscle fibers [183]. The percentage of type I fibers is known to be higher in trained endurance athletes [184], and is correlated with a higher lactate threshold [185] and negatively correlated with muscle glycogen utilization [186]. However, in untrained subjects no relationship was observed between muscle fiber type composition and RER at rest or during exercise at 55% *V*O_2max_ [187]. It is possible some degree of endurance training could be needed for type I fiber percentage to help predict RER. Future studies are needed to study the influence of training status and intensity on RER during exercise, in males and especially females as our model could not consider sex as a variable due to the lack of studies in females.

### Combined Influence of Factors

From a practical standpoint it is important to understand the net effect of modulating multiple variables at the same time, rather than just in isolation. The relative influence of each variable can be seen in Fig. 3, and predicted values when modulating two parameters at once are illustrated in Fig. 4. For example, we could expect an RER value during exercise that is ~ 0.03 units higher when consuming 1 g kg^−1^ per day of dietary fat compared with 2.5 g kg^−1^ per day (Fig. 4a), whereas increasing carbohydrate ingestion during exercise from 45 to 90 g/h would only be expected to increase RER by ~ 0.01 units (Fig. 4f). It can also be expected that someone consuming 1 g kg^−1^ dietary fat per day would have to cycle for 3 h to attain the same RER as someone consuming 2.5 g kg^−1^ per day would attain after just 1 h of cycling (Fig. 4a). For the interested reader, we have also created an online dashboard that allows users to simultaneously modulate all parameters to see the influence on predicted RER values [97].

### Other Possible Contributing Factors

Body composition has also been thought to influence RER. However, most studies have found no relationship [19, 187–190], although increases [191] and decreases [192] in RER with increasing body fat percentage have been reported. The distribution of body fat (upper vs. lower body) can influence RER during exercise via differing hormonal responses [38], potentially helping to explain some of the divergent findings. Other factors that could influence RER include cycling cadence [30, 193], hydration status [31], short-term exercise training volume [19], genetic variation [41], hyperinsulinemia [194], insulin resistance [195], daily energy and protein intake, protein supplementation during exercise, and pre-exercise glucose levels, but further investigation is needed in these areas.

### Technical Factors

Finally, a brief consideration of measurement factors is warranted. The RER represents whole-body substrate utilization, and likely underestimates the RQ at a given work rate, particularly during lower-intensity exercise, due to a dilution effect from other organs that rely more on fat oxidation [196]. At lower intensities the metabolism of non-muscle tissues has a proportionately greater influence on gas exchange and may imply a lower muscle RQ, while the relative proportion of total gas exchange derived from muscle will increase as intensity increases and result in the whole-body RER becoming closer to that of muscle [196]. Although the repeatability of RER measurements during low-intensity exercise has been shown to be very good [197], RER values at exercise intensities > 75% VO_2max_ are not reliable due to changes in the size of the bicarbonate pool [1]. Finally, this analysis was performed on group means, rather than individual values. Although not commonly performed in this manner, others have utilized a similar approach [55, 198], which could lead to a higher degree of uncertainty when predicting individual, as opposed to group mean, values. It has also been suggested that modifying factors at the group level may not accurately reflect the modifying effects at the individual level, introducing ecological bias [199]. However, the goal of this analysis was to determine which factors best explain RER during exercise and understand their relative influence, and so the risk of bias can be mitigated by accounting for potentially confounding variables in the analysis [55].

### Practical Implications

This modelling can be used by athletes and coaches to gain a better understanding of the convergence of factors influencing substrate oxidation during endurance exercise. Overall, athletes looking to increase fat oxidation during exercise should focus more on daily fat and carbohydrate intake, and to a lesser degree, pre-exercise carbohydrate intake, while being less concerned with carbohydrate ingestion during exercise, particularly as exercise duration extends. Furthermore, the easily measured and easily modifiable factors related to exercise (e.g., exercise duration and intensity, daily macronutrient intake, and pre- and peri-exercise carbohydrate intake) can only explain roughly one-third of the variation in RER during exercise, suggesting most of what dictates RER during exercise cannot be easily controlled by the athlete. However, there are other factors that can be modified such as pre-exercise meal timing and glycemic index, the type of carbohydrate ingested before and during exercise, hydration status, and cycling cadence that are not included in this model due to lack of data. The inclusion of other modifiable factors may indeed strengthen this model, but further research is required. Finally, it is important to remember that substrate oxidation is only one part of the puzzle for athletes, and just because something has little effect on RER does not mean it does not have other implications for performance and adaptations.

## Conclusion

Factors known to influence the RER during exercise, such as exercise duration and intensity, age, sex, fitness level, muscle glycogen, and daily dietary intake, together only explain ~ 60% of the variation in RER during exercise, and habitual dietary intake has a larger influence on RER than carbohydrate ingested during exercise. More research is needed on older subjects and females, particularly in relation to carbohydrate ingestion during exercise. Future studies should also investigate other potential predictors of RER including the lactate/ventilatory thresholds, training age, genetic markers, and markers of blood glucose and insulin sensitivity, which may help explain part of the remaining ~ 40% of variance in RER during exercise. Additionally, more research is needed looking at substrate oxidation beyond 4 h of exercise, especially considering the popularity of ultra-endurance events.

## Supplementary Information

Below is the link to the electronic supplementary material.

Supplementary file1 (DOCX 101 kb)

## Declarations

### Availability of data and material

Data can be made available upon request to the lead author. Correlations and model data can be explored using an online app: https://rothschild.shinyapps.io/RER_dashboard/.
