# Supplementary material for: Factors Influencing Substrate Oxidation During Submaximal Cycling: A Modelling Analysis
Source: Sports Med. 2022 Jul 12;52(11):2775–95. doi: 10.1007/s40279-022-01727-7 (PMC9585001; doi:10.1007/s40279-022-01727-7)
Supplement: Supplementary file 1 — Supplementary file1 (DOCX 101 kb) [file 40279_2022_1727_MOESM1_ESM.docx]

The following studies were included in the regression modelling:

(1-106)

1. Abildgaard J, Pedersen AT, Green CJ, Harder-Lauridsen NM, Solomon TP, Thomsen C, Juul A, Pedersen M, Pedersen JT, Mortensen OH, Pilegaard H, Pedersen BK, and Lindegaard B. Menopause is associated with decreased whole body fat oxidation during exercise. *Am J Physiol Endocrinol Metab* 304: E1227-1236, 2013.

2. Ahlborg G, Felig P, Hagenfeldt L, Hendler R, and Wahren J. Substrate turnover during prolonged exercise in man. Splanchnic and leg metabolism of glucose, free fatty acids, and amino acids. *J Clin Invest* 53: 1080-1090, 1974.

3. Arkinstall MJ, Bruce CR, Clark SA, Rickards CA, Burke LM, and Hawley JA. Regulation of fuel metabolism by preexercise muscle glycogen content and exercise intensity. *J Appl Physiol (1985)* 97: 2275-2283, 2004.

4. Ataide-Silva T, Ghiarone T, Bertuzzi R, Stathis CG, Leandro CG, and Lima-Silva AE. CHO Mouth Rinse Ameliorates Neuromuscular Response with Lower Endogenous CHO Stores. *Med Sci Sports Exerc* 48: 1810-1820, 2016.

5. Baur DA, Schroer AB, Luden ND, Womack CJ, Smyth SA, and Saunders MJ. Glucose-fructose enhances performance versus isocaloric, but not moderate, glucose. *Med Sci Sports Exerc* 46: 1778-1786, 2014.

6. Bishop NC, Walsh NP, Haines DL, Richards EE, and Gleeson M. Pre-exercise carbohydrate status and immune responses to prolonged cycling: II. Effect on plasma cytokine concentration. *Int J Sport Nutr Exerc Metab* 11: 503-512, 2001.

7. Blomstrand E, Andersson S, Hassmen P, Ekblom B, and Newsholme EA. Effect of branched-chain amino acid and carbohydrate supplementation on the exercise-induced change in plasma and muscle concentration of amino acids in human subjects. *Acta Physiol Scand* 153: 87-96, 1995.

8. Bradley NS, Heigenhauser GJ, Roy BD, Staples EM, Inglis JG, LeBlanc PJ, and Peters SJ. The acute effects of differential dietary fatty acids on human skeletal muscle pyruvate dehydrogenase activity. *J Appl Physiol (1985)* 104: 1-9, 2008.

9. Broad EM, Maughan RJ, and Galloway SD. Carbohydrate, protein, and fat metabolism during exercise after oral carnitine supplementation in humans. *Int J Sport Nutr Exerc Metab* 18: 567-584, 2008.

10. Broad EM, Maughan RJ, and Galloway SD. Effects of exercise intensity and altered substrate availability on cardiovascular and metabolic responses to exercise after oral carnitine supplementation in athletes. *Int J Sport Nutr Exerc Metab* 21: 385-397, 2011.

11. Broome SC, Braakhuis AJ, Mitchell CJ, and Merry TL. Mitochondria-targeted antioxidant supplementation improves 8 km time trial performance in middle-aged trained male cyclists. *J Int Soc Sports Nutr* 18: 58, 2021.

12. Burke LM, Angus DJ, Cox GR, Cummings NK, Febbraio MA, Gawthorn K, Hawley JA, Minehan M, Martin DT, and Hargreaves M. Effect of fat adaptation and carbohydrate restoration on metabolism and performance during prolonged cycling. *J Appl Physiol (1985)* 89: 2413-2421, 2000.

13. Burke LM, Hawley JA, Angus DJ, Cox GR, Clark SA, Cummings NK, Desbrow B, and Hargreaves M. Adaptations to short-term high-fat diet persist during exercise despite high carbohydrate availability. *Med Sci Sports Exerc* 34: 83-91, 2002.

14. Carey AL, Staudacher HM, Cummings NK, Stepto NK, Nikolopoulos V, Burke LM, and Hawley JA. Effects of fat adaptation and carbohydrate restoration on prolonged endurance exercise. *J Appl Physiol (1985)* 91: 115-122, 2001.

15. Carter SL, Rennie C, and Tarnopolsky MA. Substrate utilization during endurance exercise in men and women after endurance training. *Am J Physiol Endocrinol Metab* 280: E898-907, 2001.

16. Cole M, Coleman D, Hopker J, and Wiles J. Improved gross efficiency during long duration submaximal cycling following a short-term high carbohydrate diet. *Int J Sports Med* 35: 265-269, 2014.

17. Davison G, Callister R, Williamson G, Cooper KA, and Gleeson M. The effect of acute pre-exercise dark chocolate consumption on plasma antioxidant status, oxidative stress and immunoendocrine responses to prolonged exercise. *Eur J Nutr* 51: 69-79, 2012.

18. Davison G and Gleeson M. The effect of 2 weeks vitamin C supplementation on immunoendocrine responses to 2.5 h cycling exercise in man. *Eur J Appl Physiol* 97: 454-461, 2006.

19. De Bock K, Derave W, Ramaekers M, Richter EA, and Hespel P. Fiber type-specific muscle glycogen sparing due to carbohydrate intake before and during exercise. *J Appl Physiol* 102: 183-188, 2007.

20. Dearlove DJ, Harrison OK, Hodson L, Jefferson A, Clarke K, and Cox PJ. The Effect of Blood Ketone Concentration and Exercise Intensity on Exogenous Ketone Oxidation Rates in Athletes. *Med Sci Sports Exerc*, 2020.

21. Deli CK, Poulios A, Georgakouli K, Papanikolaou K, Papoutsis A, Selemekou M, Karathanos VT, Draganidis D, Tsiokanos A, Koutedakis Y, Fatouros IG, and Jamurtas AZ. The effect of pre-exercise ingestion of corinthian currant on endurance performance and blood redox status. *J Sports Sci* 36: 2172-2180, 2018.

22. Devries MC, Hamadeh MJ, Graham TE, and Tarnopolsky MA. 17beta-estradiol supplementation decreases glucose rate of appearance and disappearance with no effect on glycogen utilization during moderate intensity exercise in men. *J Clin Endocrinol Metab* 90: 6218-6225, 2005.

23. Devries MC, Hamadeh MJ, Phillips SM, and Tarnopolsky MA. Menstrual cycle phase and sex influence muscle glycogen utilization and glucose turnover during moderate-intensity endurance exercise. *Am J Physiol Regul Integr Comp Physiol* 291: R1120-1128, 2006.

24. Devries MC, Lowther SA, Glover AW, Hamadeh MJ, and Tarnopolsky MA. IMCL area density, but not IMCL utilization, is higher in women during moderate-intensity endurance exercise, compared with men. *Am J Physiol Regul Integr Comp Physiol* 293: R2336-2342, 2007.

25. Duhamel TA, Green HJ, Perco JG, and Ouyang J. Metabolic and sarcoplasmic reticulum Ca2+ cycling responses in human muscle 4 days following prolonged exercise. *Can J Physiol Pharmacol* 83: 643-655, 2005.

26. Duhamel TA, Green HJ, Perco JG, and Ouyang J. Comparative effects of a low-carbohydrate diet and exercise plus a low-carbohydrate diet on muscle sarcoplasmic reticulum responses in males. *Am J Physiol Cell Physiol* 291: C607-617, 2006.

27. Duhamel TA, Green HJ, Perco JG, and Ouyang J. Effects of prior exercise and a low-carbohydrate diet on muscle sarcoplasmic reticulum function during cycling in women. *J Appl Physiol (1985)* 101: 695-706, 2006.

28. Dumke CL, McBride JM, Nieman DC, Gowin WD, Utter AC, and McAnulty SR. Effect of duration and exogenous carbohydrate on gross efficiency during cycling. *J Strength Cond Res* 21: 1214-1219, 2007.

29. Edwards LM, Holloway CJ, Murray AJ, Knight NS, Carter EE, Kemp GJ, Thompson CH, Tyler DJ, Neubauer S, Robbins PA, and Clarke K. Endurance exercise training blunts the deleterious effect of high-fat feeding on whole body efficiency. *Am J Physiol Regul Integr Comp Physiol* 301: R320-326, 2011.

30. Evans M, Patchett E, Nally R, Kearns R, Larney M, and Egan B. Effect of acute ingestion of beta-hydroxybutyrate salts on the response to graded exercise in trained cyclists. *Eur J Sport Sci* 18: 376-386, 2018.

31. Fell JM, Hearris MA, Ellis DG, Moran JEP, Jevons EFP, Owens DJ, Strauss JA, Cocks M, Louis JB, Shepherd SO, and Morton JP. Carbohydrate improves exercise capacity but does not affect subcellular lipid droplet morphology, AMPK and p53 signalling in human skeletal muscle. *J Physiol*, 2021.

32. Forbes SC, Harber V, and Bell GJ. The acute effects of L-arginine on hormonal and metabolic responses during submaximal exercise in trained cyclists. *Int J Sport Nutr Exerc Metab* 23: 369-377, 2013.

33. Goedecke JH, Christie C, Wilson G, Dennis SC, Noakes TD, Hopkins WG, and Lambert EV. Metabolic adaptations to a high-fat diet in endurance cyclists. *Metabolism* 48: 1509-1517, 1999.

34. Goedecke JH, Clark VR, Noakes TD, and Lambert EV. The effects of medium-chain triacylglycerol and carbohydrate ingestion on ultra-endurance exercise performance. *Int J Sport Nutr Exerc Metab* 15: 15-27, 2005.

35. Goedecke JH, St Clair Gibson A, Grobler L, Collins M, Noakes TD, and Lambert EV. Determinants of the variability in respiratory exchange ratio at rest and during exercise in trained athletes. *Am J Physiol Endocrinol Metab* 279: E1325-1334, 2000.

36. Greer BK, White JP, Arguello EM, and Haymes EM. Branched-chain amino acid supplementation lowers perceived exertion but does not affect performance in untrained males. *J Strength Cond Res* 25: 539-544, 2011.

37. Halson SL, Lancaster GI, Achten J, Gleeson M, and Jeukendrup AE. Effects of carbohydrate supplementation on performance and carbohydrate oxidation after intensified cycling training. *J Appl Physiol (1985)* 97: 1245-1253, 2004.

38. Hamadeh MJ, Devries MC, and Tarnopolsky MA. Estrogen supplementation reduces whole body leucine and carbohydrate oxidation and increases lipid oxidation in men during endurance exercise. *J Clin Endocrinol Metab* 90: 3592-3599, 2005.

39. Hearris MA, Pugh JN, Langan-Evans C, Mann SJ, Burke L, Stellingwerff T, Gonzalez JT, and Morton JP. (13)C-glucose-fructose labeling reveals comparable exogenous CHO oxidation during exercise when consuming 120 g/h in fluid, gel, jelly chew, or coingestion. *J Appl Physiol (1985)* 132: 1394-1406, 2022.

40. Helge JW, Richter EA, and Kiens B. Interaction of training and diet on metabolism and endurance during exercise in man. *J Physiol* 492 ( Pt 1): 293-306, 1996.

41. Helge JW, Watt PW, Richter EA, Rennie MJ, and Kiens B. Fat utilization during exercise: adaptation to a fat-rich diet increases utilization of plasma fatty acids and very low density lipoprotein-triacylglycerol in humans. *J Physiol* 537: 1009-1020, 2001.

42. Helge JW, Wulff B, and Kiens B. Impact of a fat-rich diet on endurance in man: role of the dietary period. *Med Sci Sports Exerc* 30: 456-461, 1998.

43. Horton TJ, Miller EK, Glueck D, and Tench K. No effect of menstrual cycle phase on glucose kinetics and fuel oxidation during moderate-intensity exercise. *Am J Physiol Endocrinol Metab* 282: E752-E762, 2002.

44. Hulston CJ, Venables MC, Mann CH, Martin C, Philp A, Baar K, and Jeukendrup AE. Training with low muscle glycogen enhances fat metabolism in well-trained cyclists. *Med Sci Sports Exerc* 42: 2046-2055, 2010.

45. Ichinose T, Arai N, Nagasaka T, Asano M, and Hashimoto K. Impact of intensive high-fat ingestion in the early stage of recovery from exercise training on substrate metabolism during exercise in humans. *J Nutr Sci Vitaminol (Tokyo)* 58: 354-359, 2012.

46. Impey SG, Smith D, Robinson AL, Owens DJ, Bartlett JD, Smith K, Limb M, Tang J, Fraser WD, and Close GL. Leucine-enriched protein feeding does not impair exercise-induced free fatty acid availability and lipid oxidation: beneficial implications for training in carbohydrate-restricted states. *Amino acids* 47: 407-416, 2015.

47. Isacco L, Duche P, Thivel D, Meddahi-Pelle A, Lemoine-Morel S, Duclos M, and Boisseau N. Fat mass localization alters fuel oxidation during exercise in normal weight women. *Med Sci Sports Exerc* 45: 1887-1896, 2013.

48. Jacobs KA, Paul DR, Geor RJ, Hinchcliff KW, and Sherman WM. Dietary composition influences short-term endurance training-induced adaptations of substrate partitioning during exercise. *Int J Sport Nutr Exerc Metab* 14: 38-61, 2004.

49. Jacobson TL, Febbraio MA, Arkinstall MJ, and Hawley JA. Effect of caffeine co-ingested with carbohydrate or fat on metabolism and performance in endurance-trained men. *Exp Physiol* 86: 137-144, 2001.

50. Jamurtas AZ, Tofas T, Fatouros I, Nikolaidis MG, Paschalis V, Yfanti C, Raptis S, and Koutedakis Y. The effects of low and high glycemic index foods on exercise performance and beta-endorphin responses. *J Int Soc Sports Nutr* 8: 15, 2011.

51. Jansson E. On the significance of the respiratory exchange ratio after different diets during exercise in man. *Acta Physiol Scand* 114: 103-110, 1982.

52. Jansson E and Kaijser L. Effect of diet on the utilization of blood-borne and intramuscular substrates during exercise in man. *Acta Physiol Scand* 115: 19-30, 1982.

53. Jansson E and Kaijser L. Leg citrate metabolism at rest and during exercise in relation to diet and substrate utilization in man. *Acta Physiol Scand* 122: 145-153, 1984.

54. Jensen R, Ortenblad N, Stausholm MH, Skjaerbaek MC, Larsen DN, Hansen M, Holmberg HC, Plomgaard P, and Nielsen J. Heterogeneity in subcellular muscle glycogen utilisation during exercise impacts endurance capacity in men. *J Physiol* 598: 4271-4292, 2020.

55. Kang J, Robertson RJ, Denys BG, DaSilva SG, Visich P, Suminski RR, Utter AC, Goss FL, and Metz KF. Effect of carbohydrate ingestion subsequent to carbohydrate supercompensation on endurance performance. *Int J Sport Nutr* 5: 329-343, 1995.

56. Kavouras SA, Troup JP, and Berning JR. The influence of low versus high carbohydrate diet on a 45-min strenuous cycling exercise. *Int J Sport Nutr Exerc Metab* 14: 62-72, 2004.

57. Koma R and Terasawa N. Pre-Exercise Glucose Ingestion May Improve Endurance Capacity in East Asian Student Athletes with Lower Blood Glucose Response. *J Nutr Sci Vitaminol (Tokyo)* 66: 150-157, 2020.

58. Kontro H, Kozior M, Whelehan G, Amigo-Benavent M, Norton C, Carson BP, and Jakeman P. Carbohydrate and Protein Co-Ingestion Postexercise Does Not Improve Next-Day Performance in Trained Cyclists. *Int J Sport Nutr Exerc Metab*: 1-9, 2021.

59. Kuo J, Chen KW, Cheng IS, Tsai PH, Lu YJ, and Lee NY. The effect of eight weeks of supplementation with Eleutherococcus senticosus on endurance capacity and metabolism in human. *Chin J Physiol* 53: 105-111, 2010.

60. Lamont LS, McCullough AJ, and Kalhan SC. Comparison of leucine kinetics in endurance-trained and sedentary humans. *J Appl Physiol (1985)* 86: 320-325, 1999.

61. Lamont LS, McCullough AJ, and Kalhan SC. Gender differences in leucine, but not lysine, kinetics. *J Appl Physiol (1985)* 91: 357-362, 2001.

62. Lamont LS, McCullough AJ, and Kalhan SC. Gender differences in the regulation of amino acid metabolism. *J Appl Physiol (1985)* 95: 1259-1265, 2003.

63. Lane SC, Camera DM, Lassiter DG, Areta JL, Bird SR, Yeo WK, Jeacocke NA, Krook A, Zierath JR, Burke LM, and Hawley JA. Effects of sleeping with reduced carbohydrate availability on acute training responses. *J Appl Physiol (1985)* 119: 643-655, 2015.

64. Learsi SK, Ghiarone T, Silva‐Cavalcante MD, Andrade‐Souza VA, Ataide‐Silva T, Bertuzzi R, de Araujo GG, McConell G, and Lima‐Silva AE. Cycling time trial performance is improved by carbohydrate ingestion during exercise regardless of a fed or fasted state. *Scand J Med Sci Sports*, 2019.

65. Leckey JJ, Hoffman NJ, Parr EB, Devlin BL, Trewin AJ, Stepto NK, Morton JP, Burke LM, and Hawley JA. High dietary fat intake increases fat oxidation and reduces skeletal muscle mitochondrial respiration in trained humans. *FASEB J* 32: 2979-2991, 2018.

66. Margolis LM, Wilson MA, Whitney CC, Carrigan CT, Murphy NE, Hatch AM, Montain SJ, and Pasiakos SM. Exercising with low muscle glycogen content increases fat oxidation and decreases endogenous, but not exogenous carbohydrate oxidation. *Metabolism* 97: 1-8, 2019.

67. Martinez-Noguera FJ, Marin-Pagan C, Carlos-Vivas J, and Alcaraz PE. Effects of 8 Weeks of 2S-Hesperidin Supplementation on Performance in Amateur Cyclists. *Nutrients* 12, 2020.

68. McCarthy DG, Bostad W, Powley FJ, Little JP, Richards D, and Gibala MJ. Increased Cardiorespiratory Stress During Submaximal Cycling After Ketone Monoester Ingestion in Endurance-Trained Adults. *Appl Physiol Nutr Metab*, 2021.

69. McConell G, Kloot K, and Hargreaves M. Effect of timing of carbohydrate ingestion on endurance exercise performance. *Med Sci Sports Exerc* 28: 1300-1304, 1996.

70. McConell GK, Canny BJ, Daddo MC, Nance MJ, and Snow RJ. Effect of carbohydrate ingestion on glucose kinetics and muscle metabolism during intense endurance exercise. *J Appl Physiol (1985)* 89: 1690-1698, 2000.

71. McLay RT, Thomson CD, Williams SM, and Rehrer NJ. Carbohydrate loading and female endurance athletes: effect of menstrual-cycle phase. *Int J Sport Nutr Exerc Metab* 17: 189-205, 2007.

72. Mock MG, Hirsch KR, Blue MNM, Trexler ET, Roelofs EJ, and Smith-Ryan AE. Post-Exercise Ingestion of Low or High Molecular Weight Glucose Polymer Solution Does Not Improve Cycle Performance in Female Athletes. *J Strength Cond Res* 35: 124-131, 2021.

73. Nishibata I, Sadamoto T, Mutoh Y, and Miyashita M. Glucose ingestion before and during exercise does not enhance performance of daily repeated endurance exercise. *Eur J Appl Physiol Occup Physiol* 66: 65-69, 1993.

74. Nosaka N, Suzuki Y, Nagatoishi A, Kasai M, Wu J, and Taguchi M. Effect of ingestion of medium-chain triacylglycerols on moderate- and high-intensity exercise in recreational athletes. *J Nutr Sci Vitaminol (Tokyo)* 55: 120-125, 2009.

75. O'Gorman DJ, Del Aguila LF, Williamson DL, Krishnan RK, and Kirwan JP. Insulin and exercise differentially regulate PI3-kinase and glycogen synthase in human skeletal muscle. *J Appl Physiol (1985)* 89: 1412-1419, 2000.

76. Okano G, Sato Y, Takumi Y, and Sugawara M. Effect of 4h preexercise high carbohydrate and high fat meal ingestion on endurance performance and metabolism. *Int J Sports Med* 17: 530-534, 1996.

77. Oosthuyse T, Carstens M, and Millen AM. Whey or Casein Hydrolysate with Carbohydrate for Metabolism and Performance in Cycling. *Int J Sports Med* 36: 636-646, 2015.

78. Palmer GS, Borghouts LB, Noakes TD, and Hawley JA. Metabolic and performance responses to constant-load vs. variable-intensity exercise in trained cyclists. *J Appl Physiol (1985)* 87: 1186-1196, 1999.

79. Parcell AC, Ray ML, Moss KA, Ruden TM, Sharp RL, and King DS. The effect of encapsulated soluble fiber on carohydrate metabolism during exercise. *Int J Sport Nutr* 9: 13-23, 1999.

80. Pitsiladis YP and Maughan RJ. The effects of alterations in dietary carbohydrate intake on the performance of high-intensity exercise in trained individuals. *Eur J Appl Physiol Occup Physiol* 79: 433-442, 1999.

81. Ramos-Jiménez A, Hernández-Torres RP, Torres-Durán PV, Romero-Gonzalez J, Mascher D, Posadas-Romero C, and Juárez-Oropeza MA. The respiratory exchange ratio is associated with fitness indicators both in trained and untrained men: a possible application for people with reduced exercise tolerance. *Clin Med Circ Respirat Pulm Med* 2: CCRPM. S449, 2008.

82. Randell RK, Hodgson AB, Lotito SB, Jacobs DM, Rowson M, Mela DJ, and Jeukendrup AE. Variable duration of decaffeinated green tea extract ingestion on exercise metabolism. *Med Sci Sports Exerc* 46: 1185-1193, 2014.

83. Rauch HG, Hawley JA, Noakes TD, and Dennis SC. Fuel metabolism during ultra-endurance exercise. *Pflugers Arch* 436: 211-219, 1998.

84. Skillen RA, Testa M, Applegate EA, Heiden EA, Fascetti AJ, and Casazza GA. Effects of an amino acid carbohydrate drink on exercise performance after consecutive-day exercise bouts. *Int J Sport Nutr Exerc Metab* 18: 473-492, 2008.

85. Smith HA, Hengist A, Bonson DJ, Walhin JP, Jones R, Tsintzas K, Afman GH, Gonzalez JT, and Betts JA. Muscle Glycogen Utilization during Exercise after Ingestion of Alcohol. *Med Sci Sports Exerc* 53: 211-217, 2021.

86. Steffensen CH, Roepstorff C, Madsen M, and Kiens B. Myocellular triacylglycerol breakdown in females but not in males during exercise. *Am J Physiol Endocrinol Metab* 282: E634-642, 2002.

87. Stellingwerff T, Spriet LL, Watt MJ, Kimber NE, Hargreaves M, Hawley JA, and Burke LM. Decreased PDH activation and glycogenolysis during exercise following fat adaptation with carbohydrate restoration. *Am J Physiol Endocrinol Metab* 290: E380-388, 2006.

88. Stepto NK, Carey AL, Staudacher HM, Cummings NK, Burke LM, and Hawley JA. Effect of short-term fat adaptation on high-intensity training. *Med Sci Sports Exerc* 34: 449-455, 2002.

89. Stevenson EJ, Thelwall PE, Thomas K, Smith F, Brand-Miller J, and Trenell MI. Dietary glycemic index influences lipid oxidation but not muscle or liver glycogen oxidation during exercise. *Am J Physiol Endocrinol Metab* 296: E1140-1147, 2009.

90. Stocks B, Dent JR, Ogden HB, Zemp M, and Philp A. Postexercise skeletal muscle signaling responses to moderate- to high-intensity steady-state exercise in the fed or fasted state. *Am J Physiol Endocrinol Metab* 316: E230-E238, 2019.

91. Sugiura K and Kobayashi K. Effect of carbohydrate ingestion on sprint performance following continuous and intermittent exercise. *Med Sci Sports Exerc* 30: 1624-1630, 1998.

92. Tarnopolsky MA, Atkinson SA, Phillips SM, and MacDougall JD. Carbohydrate loading and metabolism during exercise in men and women. *J Appl Physiol (1985)* 78: 1360-1368, 1995.

93. Taylor C, Bartlett JD, van de Graaf CS, Louhelainen J, Coyne V, Iqbal Z, MacLaren DP, Gregson W, Close GL, and Morton JP. Protein ingestion does not impair exercise-induced AMPK signalling when in a glycogen-depleted state: implications for train-low compete-high. *Eur J Appl Physiol* 113: 1457-1468, 2013.

94. Terink R, Witkamp RF, Hopman MTE, Siebelink E, Savelkoul HFJ, and Mensink M. A 2 Week Cross-over Intervention with a Low Carbohydrate, High Fat Diet Compared to a High Carbohydrate Diet Attenuates Exercise-Induced Cortisol Response, but Not the Reduction of Exercise Capacity, in Recreational Athletes. *Nutrients* 13, 2021.

95. Tremblay J, Peronnet F, Massicotte D, and Lavoie C. Carbohydrate supplementation and sex differences in fuel selection during exercise. *Med Sci Sports Exerc* 42: 1314-1323, 2010.

96. van Zant RS and Lemon PW. Preexercise sugar feeding does not alter prolonged exercise muscle glycogen or protein catabolism. *Can J Appl Physiol* 22: 268-279, 1997.

97. Virk RS, Dunton NJ, Young JC, and Leklem JE. Effect of vitamin B-6 supplementation on fuels, catecholamines, and amino acids during exercise in men. *Med Sci Sports Exerc* 31: 400-408, 1999.

98. Vogt M, Puntschart A, Howald H, Mueller B, Mannhart C, Gfeller-Tuescher L, Mullis P, and Hoppeler H. Effects of dietary fat on muscle substrates, metabolism, and performance in athletes. *Med Sci Sports Exerc* 35: 952-960, 2003.

99. Vukovich MD, Costill DL, Hickey MS, Trappe SW, Cole KJ, and Fink WJ. Effect of fat emulsion infusion and fat feeding on muscle glycogen utilization during cycle exercise. *J Appl Physiol (1985)* 75: 1513-1518, 1993.

100. Watt MJ, Krustrup P, Secher NH, Saltin B, Pedersen BK, and Febbraio MA. Glucose ingestion blunts hormone-sensitive lipase activity in contracting human skeletal muscle. *Am J Physiol Endocrinol Metab* 286: E144-150, 2004.

101. Wenz M, Berend JZ, Lynch NA, Chappell S, and Hackney AC. Substrate oxidation at rest and during exercise: effects of menstrual cycle phase and diet composition. *J Physiol Pharmacol* 48: 851-860, 1997.

102. Whitley HA, Humphreys SM, Campbell IT, Keegan MA, Jayanetti TD, Sperry DA, MacLaren DP, Reilly T, and Frayn KN. Metabolic and performance responses during endurance exercise after high-fat and high-carbohydrate meals. *J Appl Physiol (1985)* 85: 418-424, 1998.

103. Yeo WK, Lessard SJ, Chen Z-P, Garnham AP, Burke LM, Rivas DA, Kemp BE, and Hawley JA. Fat adaptation followed by carbohydrate restoration increases AMPK activity in skeletal muscle from trained humans. *J Appl Physiol* 105: 1519-1526, 2008.

104. Zachwieja JJ, Costill DL, and Fink WJ. Carbohydrate ingestion during exercise: effects on muscle glycogen resynthesis after exercise. *Int J Sport Nutr* 3: 418-430, 1993.

105. Zajac A, Poprzecki S, Maszczyk A, Czuba M, Michalczyk M, and Zydek G. The effects of a ketogenic diet on exercise metabolism and physical performance in off-road cyclists. *Nutrients* 6: 2493-2508, 2014.

106. Zehnder M, Christ ER, Ith M, Acheson KJ, Pouteau E, Kreis R, Trepp R, Diem P, Boesch C, and Decombaz J. Intramyocellular lipid stores increase markedly in athletes after 1.5 days lipid supplementation and are utilized during exercise in proportion to their content. *Eur J Appl Physiol* 98: 341-354, 2006.
